# Dimethyl 5,5′-methyl­enebis(2-hy­droxy­benzoate)

**DOI:** 10.1107/S1600536812015760

**Published:** 2012-04-18

**Authors:** Samuel Guieu, Paula Brandão, João Rocha, Artur M. S. Silva

**Affiliations:** a University of Aveiro, QOPNA, Department of Chemistry, 3810-193 Aveiro, Portugal; b University of Aveiro, CICECO, Department of Chemistry, 3810-193 Aveiro, Portugal

## Abstract

In the title compound, C_17_H_16_O_6_, the two methyl salicylate moieties are related by crystallographic twofold rotational symmetry with the two benzene rings close to being perpendicular [inter-ring dihedral angle = 86.6 (8)°]. Intra­molecular phenolic O—H⋯O hydrogen bonds with carboxyl O-atom acceptors are present, with these groups also involved in centrosymmetric cyclic inter­molecular O—H⋯O hydrogen-bonding associations [graph set *R*_2_^2^(4)], giving infinite chains extending across (101).

## Related literature

For the chemistry and applications of methyl­ene bis­phenol derivatives, see: Ogata *et al.* (1975 ▶); Méric *et al.* (1993 ▶); Shrestha *et al.* (2007 ▶); Cameron *et al.* (2002 ▶). For the preparation, see: Cushman & Kanamathareddy (1990 ▶); Méric *et al.* (1993 ▶). For the structures of similar compounds, see: Lu *et al.* (2011 ▶); Zhang *et al.* (2009 ▶); Liu *et al.* (2009 ▶). For graph-set analysis, see Etter *et al.* (1990 ▶). For bond-length data, see: Allen *et al.* (1987 ▶).
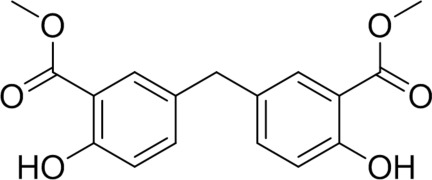


## Experimental

### 

#### Crystal data

C_17_H_16_O_6_
*M* *_r_* = 316.31
Monoclinic, 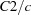

*a* = 20.4168 (13) Å
*b* = 4.9300 (3) Å
*c* = 15.5470 (12) Å
β = 111.290 (3)°
*V* = 1458.08 (17) Å^3^
*Z* = 4
Mo *K*α radiation
μ = 0.11 mm^−1^
*T* = 150 K
0.38 × 0.30 × 0.24 mm

#### Data collection

Bruker SMART CCD-detector diffractometer
Absorption correction: multi-scan (*SADABS*; Bruker, 2008 ▶) *T*_min_ = 0.953, *T*_max_ = 0.970
8440 measured reflections
1756 independent reflections
1568 reflections with *I* > 2σ(*I*)
*R*_int_ = 0.025

#### Refinement

*R*[*F* ^2^ > 2σ(*F* ^2^)] = 0.040
*wR*(*F* ^2^) = 0.113
*S* = 0.96
1756 reflections
109 parameters
H atoms treated by a mixture of independent and constrained refinement
Δρ_max_ = 0.23 e Å^−3^
Δρ_min_ = −0.33 e Å^−3^

### 

Data collection: *SMART* (Bruker, 2008 ▶); cell refinement: *SAINT* (Bruker, 2008 ▶); data reduction: *SAINT*; program(s) used to solve structure: *SHELXS97* (Sheldrick, 2008 ▶); program(s) used to refine structure: *SHELXL97* (Sheldrick, 2008 ▶); molecular graphics: *PLATON* (Spek, 2009 ▶); software used to prepare material for publication: *SHELXL97*.

## Supplementary Material

Crystal structure: contains datablock(s) global, I. DOI: 10.1107/S1600536812015760/zs2198sup1.cif

Structure factors: contains datablock(s) I. DOI: 10.1107/S1600536812015760/zs2198Isup2.hkl

Supplementary material file. DOI: 10.1107/S1600536812015760/zs2198Isup3.cml

Additional supplementary materials:  crystallographic information; 3D view; checkCIF report

## Figures and Tables

**Table 1:** Hydrogen-bond geometry (Å, °)

*D*—H⋯*A*	*D*—H	H⋯*A*	*D*⋯*A*	*D*—H⋯*A*
O1—H1⋯O2	0.87 (2)	1.87 (2)	2.6457 (12)	147 (2)
O1—H1⋯O2^i^	0.87 (2)	2.32 (1)	3.0067 (11)	134 (9)

Symmetry code: (i) 
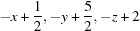
.

**Fig. 1.**
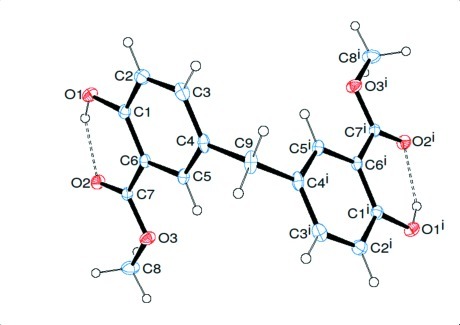
The molecular structure of the title compound showing atom numbering and displacement ellipsoids drawn at the 30% probability level. The intramolecular hydrogen bonds are shown as dashed lines. Symmetry code: (i) -x + 1, y, -z + 1/2.

**Fig. 2.**
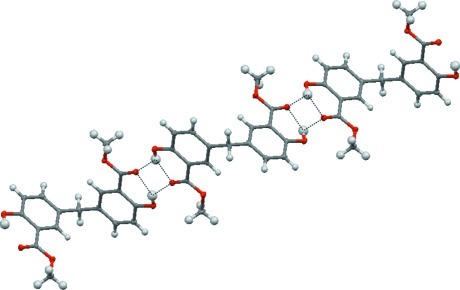
The one-dimensional hydrogen-bonded chains in the title compound, with hydrogen bonds shown as dashed lines. Displacement ellipsoids are drawn at the 30% probability level.

**Fig. 3.**
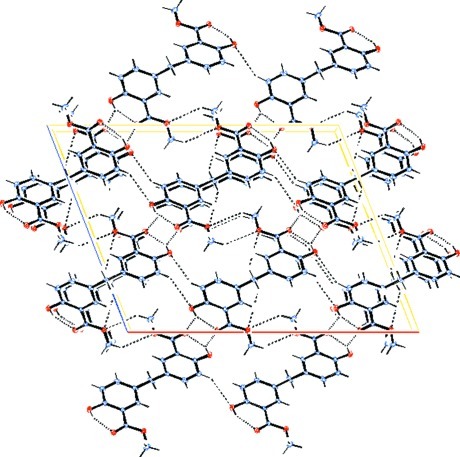
The packing of the title compound in the unit cell viewed down the b axis, with hydrogen bonds and other intermolecular interactions shown as dashed lines.

## supplementary crystallographic information

### Comment

The title compound C_17_H_16_O_6_ was first reported as a building block for polymer synthesis (Ogata *et al.*, 1975). It is a useful precursor for organic polymers, metal-organic frameworks, cage compounds (Méric *et al.*, 1993) and biologically active compounds (Shrestha *et al.*, 2007; Cameron *et al.*, 2002). In the title compound (Fig. 1), the two methyl salicylate moieties are related by crystallographic twofold rotational symmetry with the two phenyl rings close to perpendicular [inter-ring dihedral angle = 86.6 (8)°]. Bond lengths and angles are within normal ranges (Allen *et al.*, 1987). Intramolecular phenolic O—H···O hydrogen bonds with carboxyl O-atom acceptors are present, with these groups also involved in centrosymmetric cyclic intermolecular hydrogen-bonding associations [graph set *R*^2^_2_(4) (Etter *et al.*, 1990)], making the ester group essentially coplanar with the phenyl ring [torsion angle C1—C6—C7—O3, 178.64 (9)°]. The molecules are involved in centrosymmetric cyclic intermolecular phenolic O—H···O_carboxyl_ hydrogen-bonding associations [graph set *R*^2^_2_(4) giving infinite chains extending across (101) (Figs. 2, 3).

### Experimental

The title compound was prepared in two steps starting with salicylic acid. 5,5'-Methylenebis(salicylic acid) was prepared according to a known procedure (Cushman *et al.*, 1990), and was then esterified with methanol and a catalytic amount of sulfuric acid (Méric *et al.*, 1993). Slow evaporation of a saturated solution in dichloromethane gave single crystals suitable for X-ray diffraction.

### Refinement

The phenolic H-atom (H1) was located in a difference Fourier map and both positional and isotropic displacement parameters were refined. All other H-atoms were placed in geometrically idealized positions and refined using a riding model with C—H = 0.95 Å (aromatic), 0.98 Å (methylene) or 0.97 Å (methyl) and *U*_iso_(H) = 1.2*U*_eq_(C) (aromatic or methylene) or *U*_iso_(H) = 1.5*U*_eq_(C) (methyl).

### Crystal data

**Table:**

C_17_H_16_O_6_	*F*(000) = 664
*M**_r_* = 316.31	*D*_x_ = 1.441 Mg m^−^^3^
Monoclinic, *C*2/*c*	Mo *K*α radiation, λ = 0.71073 Å
Hall symbol: -C 2yc	Cell parameters from 8440 reflections
*a* = 20.4168 (13) Å	θ = 2.8–27.9°
*b* = 4.9300 (3) Å	µ = 0.11 mm^−^^1^
*c* = 15.5470 (12) Å	*T* = 150 K
β = 111.290 (3)°	Block, colourless
*V* = 1458.08 (17) Å^3^	0.38 × 0.30 × 0.24 mm
*Z* = 4	

### Data collection

**Table:**

Bruker SMART CCD-detector diffractometer	1756 independent reflections
Radiation source: fine-focus sealed tube	1568 reflections with *I* > 2σ(*I*)
Graphite monochromator	*R*_int_ = 0.025
ω and φ scans	θ_max_ = 27.9°, θ_min_ = 4.1°
Absorption correction: multi-scan (*SADABS*; Bruker, 2008)	*h* = −26→21
*T*_min_ = 0.953, *T*_max_ = 0.970	*k* = −6→6
8440 measured reflections	*l* = −19→20

### Refinement

**Table:**

Refinement on *F*^2^	Primary atom site location: structure-invariant direct methods
Least-squares matrix: full	Secondary atom site location: difference Fourier map
*R*[*F*^2^ > 2σ(*F*^2^)] = 0.040	Hydrogen site location: inferred from neighbouring sites
*wR*(*F*^2^) = 0.113	H atoms treated by a mixture of independent and constrained refinement
*S* = 0.96	*w* = 1/[σ^2^(*F*_o_^2^) + (0.0724*P*)^2^ + 0.9516*P*] where *P* = (*F*_o_^2^ + 2*F*_c_^2^)/3
1756 reflections	(Δ/σ)_max_ = 0.049
109 parameters	Δρ_max_ = 0.23 e Å^−^^3^
0 restraints	Δρ_min_ = −0.33 e Å^−^^3^

### Special details

**Table:**

Geometry. All s.u.'s (except the s.u. in the dihedral angle between two l.s. planes) are estimated using the full covariance matrix. The cell s.u.'s are taken into account individually in the estimation of s.u.'s in distances, angles and torsion angles; correlations between s.u.'s in cell parameters are only used when they are defined by crystal symmetry. An approximate (isotropic) treatment of cell s.u.'s is used for estimating s.u.'s involving l.s. planes.
Refinement. Refinement of *F*^2^ against ALL reflections. The weighted *R*-factor *wR* and goodness of fit *S* are based on *F*^2^, conventional *R*-factors *R* are based on *F*, with *F* set to zero for negative *F*^2^. The threshold expression of *F*^2^ > 2σ(*F*^2^) is used only for calculating *R*-factors(gt) *etc*. and is not relevant to the choice of reflections for refinement. *R*-factors based on *F*^2^ are statistically about twice as large as those based on *F*, and *R*- factors based on ALL data will be even larger.

### Fractional atomic coordinates and isotropic or equivalent isotropic displacement parameters (Å^2^)

**Table:**

	*x*	*y*	*z*	*U*_iso_*/*U*_eq_	Occ. (<1)
H1	0.2434 (10)	1.009 (5)	0.9251 (15)	0.070 (6)*	
O2	0.18996 (4)	1.12860 (16)	0.99304 (5)	0.0260 (2)	
O1	0.24736 (4)	0.89366 (17)	0.88455 (6)	0.0265 (2)	
O3	0.08604 (4)	0.98089 (17)	0.99095 (6)	0.0284 (2)	
C5	0.07384 (5)	0.6081 (2)	0.85532 (7)	0.0212 (2)	
H5	0.0372	0.6288	0.8789	0.025*	
C6	0.13366 (5)	0.7717 (2)	0.89054 (7)	0.0189 (2)	
C2	0.18118 (6)	0.5483 (2)	0.78810 (8)	0.0269 (3)	
H2	0.2178	0.5240	0.7648	0.032*	
C7	0.14042 (5)	0.9762 (2)	0.96221 (7)	0.0196 (2)	
C3	0.12105 (6)	0.3904 (2)	0.75449 (8)	0.0277 (3)	
H3	0.1170	0.2602	0.7078	0.033*	
C1	0.18813 (5)	0.7425 (2)	0.85599 (7)	0.0212 (2)	
C4	0.06631 (5)	0.4174 (2)	0.78730 (7)	0.0238 (2)	
C9	0.0000	0.2479 (3)	0.7500	0.0296 (4)	
H9A	−0.0029	0.1295	0.7999	0.036*	0.50
H9B	0.0029	0.1295	0.7001	0.036*	0.50
C8	0.09263 (7)	1.1785 (3)	1.06230 (9)	0.0350 (3)	
H8A	0.0510	1.1700	1.0796	0.053*	
H8B	0.0966	1.3604	1.0393	0.053*	
H8C	0.1347	1.1389	1.1165	0.053*	

### Atomic displacement parameters (Å^2^)

**Table:**

	*U*^11^	*U*^22^	*U*^33^	*U*^12^	*U*^13^	*U*^23^
O2	0.0226 (4)	0.0255 (4)	0.0290 (4)	−0.0081 (3)	0.0084 (3)	−0.0051 (3)
O1	0.0221 (4)	0.0265 (4)	0.0335 (5)	−0.0035 (3)	0.0132 (3)	−0.0013 (3)
O3	0.0247 (4)	0.0324 (5)	0.0315 (4)	−0.0085 (3)	0.0142 (3)	−0.0101 (3)
C5	0.0180 (4)	0.0180 (5)	0.0235 (5)	0.0006 (4)	0.0027 (4)	0.0024 (4)
C6	0.0178 (4)	0.0171 (5)	0.0188 (5)	0.0007 (3)	0.0031 (4)	0.0023 (4)
C2	0.0289 (5)	0.0259 (5)	0.0266 (5)	0.0049 (4)	0.0110 (4)	0.0009 (4)
C7	0.0175 (4)	0.0200 (5)	0.0191 (5)	−0.0013 (3)	0.0043 (4)	0.0025 (4)
C3	0.0335 (6)	0.0207 (5)	0.0235 (5)	0.0050 (4)	0.0039 (4)	−0.0024 (4)
C1	0.0200 (5)	0.0199 (5)	0.0218 (5)	0.0014 (4)	0.0052 (4)	0.0045 (4)
C4	0.0227 (5)	0.0157 (5)	0.0242 (5)	0.0020 (4)	−0.0021 (4)	0.0026 (4)
C9	0.0247 (7)	0.0162 (7)	0.0350 (8)	0.000	−0.0045 (6)	0.000
C8	0.0396 (7)	0.0382 (7)	0.0338 (6)	−0.0085 (5)	0.0211 (5)	−0.0124 (5)

### Geometric parameters (Å, º)

**Table:**

O2—C7	1.2104 (13)	C2—C1	1.3938 (15)
O1—C1	1.3508 (13)	C2—H2	0.9500
O1—H1	0.87 (2)	C3—C4	1.3932 (17)
O3—C7	1.3390 (12)	C3—H3	0.9500
O3—C8	1.4455 (14)	C4—C9	1.5157 (13)
C5—C4	1.3811 (15)	C9—C4^i^	1.5157 (13)
C5—C6	1.3989 (14)	C9—H9A	0.9900
C5—H5	0.9500	C9—H9B	0.9900
C6—C1	1.4071 (14)	C8—H8A	0.9800
C6—C7	1.4715 (14)	C8—H8B	0.9800
C2—C3	1.3857 (16)	C8—H8C	0.9800
			
C1—O1—H1	106.9 (13)	O1—C1—C6	123.74 (10)
C7—O3—C8	114.23 (8)	C2—C1—C6	118.82 (10)
C4—C5—C6	122.07 (10)	C5—C4—C3	117.65 (10)
C4—C5—H5	119.0	C5—C4—C9	120.26 (10)
C6—C5—H5	119.0	C3—C4—C9	122.08 (9)
C5—C6—C1	119.36 (9)	C4—C9—C4^i^	113.07 (12)
C5—C6—C7	121.34 (9)	C4—C9—H9A	109.0
C1—C6—C7	119.30 (9)	C4^i^—C9—H9A	109.0
C3—C2—C1	120.27 (10)	C4—C9—H9B	109.0
C3—C2—H2	119.9	C4^i^—C9—H9B	109.0
C1—C2—H2	119.9	H9A—C9—H9B	107.8
O2—C7—O3	122.15 (9)	O3—C8—H8A	109.5
O2—C7—C6	124.07 (9)	O3—C8—H8B	109.5
O3—C7—C6	113.78 (8)	H8A—C8—H8B	109.5
C2—C3—C4	121.81 (10)	O3—C8—H8C	109.5
C2—C3—H3	119.1	H8A—C8—H8C	109.5
C4—C3—H3	119.1	H8B—C8—H8C	109.5
O1—C1—C2	117.44 (9)		
			
C8—O3—C7—O2	−1.07 (15)	C3—C4—C5—C6	0.43 (15)
C8—O3—C7—C6	179.17 (9)	C9—C4—C5—C6	−178.93 (9)
O1—C1—C2—C3	−178.69 (10)	C3—C4—C9—C4^i^	−123.32 (10)
C6—C1—C2—C3	1.01 (16)	C5—C4—C9—C4^i^	56.01 (11)
O1—C1—C6—C5	178.95 (10)	C4—C5—C6—C1	0.00 (16)
O1—C1—C6—C7	−0.29 (15)	C4—C5—C6—C7	179.24 (10)
C2—C1—C6—C5	−0.73 (15)	C1—C6—C7—O2	1.60 (16)
C2—C1—C6—C7	−179.97 (10)	C1—C6—C7—O3	−178.64 (9)
C1—C2—C3—C4	−0.58 (17)	C5—C6—C7—O2	−177.63 (10)
C2—C3—C4—C5	−0.15 (16)	C5—C6—C7—O3	2.13 (14)
C2—C3—C4—C9	179.20 (10)		

Symmetry code: (i) −*x*, *y*, −*z*+3/2.

### Hydrogen-bond geometry (Å, º)

**Table:**

*D*—H···*A*	*D*—H	H···*A*	*D*···*A*	*D*—H···*A*
O1—H1···O2	0.87 (2)	1.87 (2)	2.6457 (12)	147 (2)
O1—H1···O2^ii^	0.87 (2)	2.32 (1)	3.0067 (11)	134 (9)

Symmetry code: (ii) −*x*+1/2, −*y*+5/2, −*z*+2.
